# Sociopolitical Diagnostic Tools to Understand National and Local Response Capabilities and Vulnerabilities to Epidemics and Guide Research into How to Improve the Global Response to Pathogens

**DOI:** 10.3390/pathogens12081023

**Published:** 2023-08-08

**Authors:** Samuel R. Friedman, David C. Perlman, Dimitrios Paraskevis, Justin Feldman

**Affiliations:** 1 NYU Grossman School of Medicine, New York, NY 10016, USA; 2 Infectious Diseases, Mount Sinai Beth Israel, Icahn School of Medicine at Mount Sinai, New York, NY 10029, USA; david.perlman@mountsinai.org; 3 Department of Hygiene, Epidemiology and Medical Statistics, Medical School, National and Kapodistrian University of Athens, 11527 Athens, Greece; dparask@med.uoa.gr; 4 Visiting Scientist, Harvard FXB Center for Health and Human Rights, Cambridge, MA 02138, USA; jfeldman@hsph.harvard.edu

**Keywords:** HIV, SARS-CoV-2, sociopolitical diagnostics, public health and medical funding, social influence networks, social cohesion and trust, stigmatization

## Abstract

The AIDS and COVID-19 pandemics demonstrated that nations at similar economic development levels varied widely in their capacity to protect the health of their residents. For AIDS, Britain and Australia brought gay representatives into official counsels and adopted harm reduction far more rapidly than the United States or Spain, and East African countries responded more effectively than South Africa or the Democratic Republic of the Congo. National responses to COVID-19 varied widely, with New Zealand, China, and Vietnam more effective than Italy, Brazil, or the United States. Further, as phylogenetic research has demonstrated, these pandemics spread from one country to another, with those that responded poorly acting as sources for mutations and potentially sources of transmission to countries with more effective responses. Many observers expressed surprise at the poor responses of the United States to COVID-19, but in retrospect the cutbacks in public health funding at state and national levels made it clear that this was a predictable weakness even in addition to the political vacillations that crippled the US and Brazilian responses. In a time of global sociopolitical and climate instability, it is important to measure and conduct research into spatial and time variations in 1. public health and medical funding, 2. social influence networks, social cohesion and trust, and stigmatization, 3. income inequality, 4. social conflict, and 5. other factors that affect responsiveness to pandemics.

## 1. Introduction

The AIDS and COVID-19 pandemics demonstrated that nations at similar economic development levels varied widely in the degree to which they effectively protected the health of their residents. In part, this resulted from differences in political agendas and political policies, but responses and their public health implications also depended on a wide range of social and economic structures and processes.

Many observers expressed surprise at the poor responses of the United States to COVID-19. In retrospect, prior cutbacks in public health funding at state and national levels, the chaotic decisions of for-profit and nonprofit health care systems, and known associations of racial/ethnic and economic inequality and health outcomes suggest that this was in part a predictable weakness. In addition, in the United States (as well as in other countries including Brazil, Britain, and India), unpredicted political vacillations and policies led to large numbers of avoidable deaths and lasting disease.

Developing measures that can assist governments and publics to predict and anticipate the effectiveness of response to pandemics, and thus perhaps help them to prepare better, will not be easy. These difficulties are clearly illustrated by the failures of one effort to do this: the Global Health Security Index (GHS Index) [1]. This is evident from the GHS Overall Index scores in 2019 (pre-pandemic) for three of the countries that had some of the worst mortality rates during COVID-19 (up to March 2023). The score for the United States was 1 (the best of 195 countries), for the United Kingdom 2, and for Brazil 22. By comparison, the scores for four countries that had among the lowest mortality rates were New Zealand 35, Vietnam 50, China 51, and Uganda 63. Clearly, although these scores were developed by experts in the field and included assessment of many specific indicators, better methods are needed. Relevant needs based on the HIV/AIDS and COVID-19 pandemic experiences have been discussed in a number of papers [2,3,4,5].

The COVID-19 pandemic is by no means the first one in which economic factors and political leadership failed to explain much of the variation in outcomes among nations. We will illustrate this with examples from the HIV/AIDS pandemic before returning to COVID-19. The countries shown for comparison have been chosen to show that outcomes vary in ways that existing measures do not explain, and they are by no means an exhaustive list. Some countries with interesting histories in one or both pandemics, such as Sweden and the Netherlands, are not included, for example.

## 2. AIDS

Table 1 shows that considerable variation exists in HIV incidence and prevalence. These differences are by no means adequately explained either by “founder” data from early in the HIV pandemic nor by relative income levels. South Africa’s epidemic began well after Uganda’s, and South Africa is more prosperous than Uganda, yet HIV rates are considerably higher in South Africa.

Available data suggest only partial explanations for these differences. Importantly, data on the social networks and social norms of various communities in different countries are very limited, as are data on stigma towards social and racial/ethnic groups and data on the extent to which the norms about sex and drug use vary across age cohorts in those communities. Data on public attitudes towards science and towards public officials are available, if at all, primarily through national surveys that do not include sufficient information to predict how various communities will respond to a pandemic. It should be noted that the Global Health Security Index does not include measures of most of these variables. Yet, for HIV/AIDS and many other diseases, these issues are central. Economic data, data on education levels and school budgets, on the structures of politics and issues of political contention, and on health personnel and budgets were included in the GHS Index, and are more available, but did not allow the GHS Index to predict COVID outcomes. The brief descriptions below of what can be explained about a few HIV epidemics show the limitations of current diagnostic tools and research.

Although comparable data are not available at a population level for Russia and Ukraine, both faced prolonged periods of economic collapse during the 1990s. They also faced youth disillusionment and continued increases in injected drug use (which had begun to increase some years before) [7] and sex work, with HIV and other diseases spreading among people who inject drugs and sex workers in each country. Soros foundation-funded harm reduction programs started in both countries on a small scale during the 1990s. Russia repressed harm reduction efforts, stigmatized affected individuals, and maintained a ban on methadone programs [8]. Beginning in about 2000 [9], Ukraine allowed donor-funded harm reduction to expand and to some degree encouraged it, which led to behavioral risk reduction [10]. HIV among Ukrainian PWID began to decline for about a dozen years. According to World Health Organization country fact sheets, the HIV prevalence levels among people who inject drugs in 2021 were 26% in Russia and 20.9% in Ukraine (https://cfs.hivci.org/index.html accessed on 3 March 2023). It is important to understand what we do not know about Ukraine and Russia that may be of central importance in their epidemics. We know little or nothing about the drug risk networks and sexual networks of people who inject drugs or sex workers during the 1990s, nor about how they changed in the 2000s. We have very little understanding of why Russian politics developed in ways that led to the suppression of harm reduction programs whereas Ukrainian politics did not lead in this direction. Although we know that stigma towards people who inject drugs, sex workers, and gay men was high in both Russia and Ukraine in the 1990s, we do not know why the Russian government acted to maintain policies which reinforced discrimination and these stigmas whereas the Ukrainian government moved in the other direction (to an extent). We would add that the Russian war in Ukraine has created conditions likely to increase transmission both of HIV and SARS-CoV-2 in both countries in both the short and long run, and to create political conditions that could make it difficult to implement effective public health and medical actions [2].

Even though British Prime Minister Thatcher was every bit as politically conservative as President Reagan of the United States, Britain and Australia brought gay representatives into official counsels and adopted harm reduction far more rapidly than Reagan-era (or than Bush Sr. or Clinton-era) United States or than Spain. They were also quicker to establish syringe service programs than the United States. Australia also funded organizations of people who inject drugs, in every state. As we can see in Table 1, Australia has controlled the spread of HIV much better than has the United States. (Comparable data for Britain are not available, but what does exist suggests that Britain also has done much better than the United States).

In Uganda, HIV outbreaks spread beyond isolated contexts in the early 1980s, in part due to internal war. As the war ended, the new government responded with its appropriate ABC program (Abstinence; if not, Be faithful; if not, use Condoms.) In South Africa, guerilla warfare and mass struggle against apartheid led to formal democratization in 1993, but this was followed by privatizations and cutbacks in the health sector [11,12], little attention to a spreading HIV epidemic, and then to AIDS denialism (which was met with mass protest). These processes and their epidemiology have been widely described in the literature. Thornton’s *Unimagined Community: Sex, Networks and AIDS in Uganda and South Africa* [13] summarizes much of the situation. Nonetheless, comparable data on norms, networks, trust in science and public officials, and the like do not exist for these countries. Thus, we have only a partial understanding either of the politics of the pandemic in the two countries or of why the HIV pandemic was limited much sooner in Uganda than in South Africa.

## 3. COVID-19

**Figure 1:**
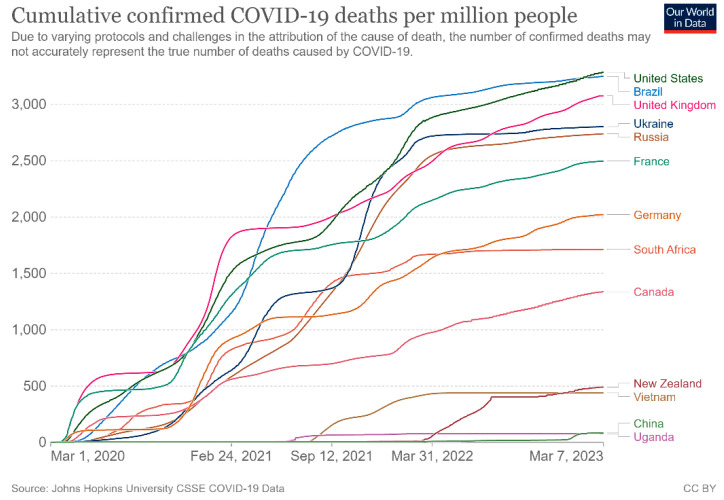
COVID-19 death rates for selected countries (Source: Our World in Data, https://ourworldindata.org/, downloaded 8 March 2023).

National responses to COVID-19 varied widely, with those of New Zealand, China, and Vietnam being more effective than the United Kingdom, Brazil, or the United States (See Figure 1; and Table 2). As with HIV, neither the time at which the outbreak began nor the economic development level of the country explains the relative rankings. Although the degree of rejection of, and indeed contempt for, scientific findings by Presidents Bolsonaro and Trump may explain the high mortality rates in Brazil and the United States to some degree, the continued considerable increase in US mortality during the Biden presidency suggests that other factors also need to be considered [14].

## 4. No Country Is an Island: One Country’s Failure Creates Problems for Other Countries

Further, as phylogenetic research has demonstrated, viruses and thus pandemics spread from one country to another, with those countries that respond poorly acting as sources for new strains with mutations potentially affecting transmissibility, pathogenicity, or vaccine-induced protection, and potentially as sources of transmission to countries with more effective responses [15,16,17,18,19,20,21,22,23,24,25,26] and to countries linked by the movement of commodities and people. Diagnostic tools to understand how to reduce such spread and how to optimize international cooperation in response to it are needed. Such tools need to assess business, military, migration, and tourist travel among nations, capabilities to prevent and detect cross-border disease transmission, and the willingness and ability to share accurate and relevant data and specimens relevant to epidemiology and interventions. They also should consider how nations might respond to efforts by international agencies, public health and medical assistance organizations, and activist groups (such as ACT UP and the International Harm Reduction Association in the first decades of the AIDS pandemic) to assist them during a pandemic.

## 5. The Value of Developing Sociopolitical Diagnostic Tools for Pandemic Planning and Response

Sociopolitical diagnostic tools can help improve responses to pandemics in several ways. First, such tools may show that a given country has weaknesses, and this may help the people who live there to take action to eliminate them. Second, when a pandemic arises, these tools might help officials and/or residents of the country to take ameliorative action. Third, since an outbreak in one country poses threats to other countries, knowledge that a given country is unlikely to respond well may be valuable in helping other countries defend themselves and others.

Developing such tools will not be easy. For one thing, validating their worth will depend in part on seeing how they perform under the stress of pandemics. For another, there are a large number of theoretical frameworks and paradigms in the social sciences, and they are likely to suggest a wide variety of different measures that will need to be evaluated.

Greer et al. [27], for example, analyzed cross-national COVID-19 politics during the first year of the pandemic using standard comparative political science concepts of “regime type” (which categorized regimes in terms of the extent to which they are authoritarian, majoritarian, centralized, federalist, and so forth), their social policies, and their public health capacity. They found these measures helped them understand how a number of countries reacted to the pandemic.

Friedman and his colleagues have presented a Big Events model that integrates some of the other structures and processes that need to be considered in conducting research and social surveillance on zoonotic outbreaks [2,28] such as HIV and SARS-CoV-2. These include pre-existing power and economic divides (such as those around structural racism and within workplaces), differences in industrial and occupational structures, the strength or weakness of pre-existing public health and medical systems [29], along with aspects of culture such as the strength of norms and values around issues such as solidarity and altruism compared with individualism, and much else. They also include measures of social movement strength, goals, and outcomes, as shown by mass mobilizations against masking and vaccination in some countries in the case of COVID-19 (or by ACT UP, Treatment Action Campaign, and harm reduction direct action for HIV prevention and care).

Additional variables that might be considered include the extent to which a given nation is in debt, the extent to which its decision-makers have to take account of the reactions of international debt-holders [30,31,32,33,34], the characteristics of its agricultural system [35], and the extent to which internal constraints limit the independence of decision-makers. These and other factors and their impacts on HIV, hepatitis C, opioid misuse, and overdose epidemics in various countries are discussed by Perlman and Jordan in the context of syndemic theory [36].

In addition, after the initial COVID-19 pandemic public health responses, the extent to which public health departments or even state or city chief executives could act on their own to declare public health emergencies was reduced in some states within the United States, and perhaps elsewhere. This highlights the importance of developing both diagnostic tools and analytic tools to understand the political complexity of political responses to pandemics.

## 6. Conclusions: Sociopolitical Diagnostics to Improve the Global Response to Pathogens

**Figure 2:**
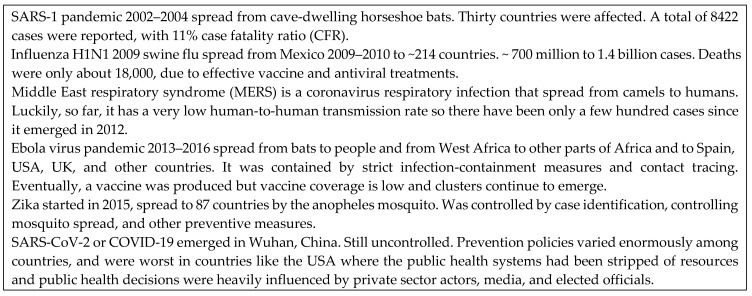
Pandemics in the 21st Century [37].

The 21st century has already had a number of pandemics and potential pandemics (see Figure 2). The history of both AIDS and COVID-19 shows that nations varied greatly in the effectiveness of their response. Existing research is suggestive, but has considered only a limited range of potential variables that may be important.

This means that WHO, national and local governments (including public health agencies), researchers, the media, and the public lack an adequate scientific base on which to base decision-making and public messaging. Research is thus sorely needed.

Specifically, we call for research and institutional development to establish and then to study sociopolitical diagnostic systems that develop and validate a broad range of sociopolitical and cultural diagnostic measures that can be used to help understand which countries are most at risk and how to strengthen their ability to weather pandemics. Such measures should include patterns of public health and medical funding, social influence networks in various population subgroups, social norms towards altruism and solidarity, patterns of social cohesion and schism, stigmatizations of and in different subpopulations, patterns of social conflict, and attitudes and political approaches to the validity of science. We are sure that this list misses many important variables, and call upon researchers and funders to improve it as we conduct the needed research.

## Figures and Tables

**Table 1:** HIV statistics, 2015 (UNAIDS) [6].

Country/Region	Adult (Ages 15–49) in Percent	HIV Incidence per 1000 Person-Years
South Africa	19.2	6.65
Uganda	6.8	1.99
Ukraine	0.9	0.25
Brazil	0.5	0.23
United States	0.5	0.12
Spain	0.4	0.09
Australia	0.1	0.05

**Table 2:** Excess mortality during 2020 through 2022 for selected countries *.

Country	cum_p_proj_all_ages **
Brazil	20.17
China *	
Great Britain	10
India *	
New Zealand	−0.44
Russia	24.43
South Africa	18.61
Uganda *	
Ukraine *	
United States	14.23
Vietnam *	

Source: Estimates of Excess Mortality Associated With COVID-19 Pandemic (as of 5 April 2023). Geneva: World Health Organization, 2023. * Earlier data from WHO for 2020–2021, using different methodology, reported that China and Vietnam had fewer projected deaths during this period, that Uganda had very low excess mortality, but that Ukraine and India had excess mortality rates worse than the United States though better than Russia. ** The cum_p_proj_all_ages is the percentage difference between the cumulative number of deaths since 1 January 2020 and the cumulative projected deaths for the same period based on previous years. The reported number might not include all deaths that occurred, due to incomplete coverage and delays in reporting.
